# The Norwegian childhood cancer biobank

**DOI:** 10.1002/cnr2.1555

**Published:** 2021-09-20

**Authors:** Johanne U. Hermansen, Dorota M. Wojcik, Nina Robinson, Jens Pahnke, Hans Kristian Haugland, Ann Helen Jamtøy, Trond Flægstad, Hanne Halvorsen, Bendik Lund, Lars O. Baumbusch, Monica C. Munthe‐Kaas

**Affiliations:** ^1^ Department of Oncology, Division of Pediatric and Adolescent Medicine Oslo University Hospital Rikshospitalet Oslo Norway; ^2^ Department of Pediatric Research, Division of Pediatric and Adolescent Medicine Oslo University Hospital Rikshospitalet Oslo Norway; ^3^ Department of Pediatrics Haukeland University Hospital Bergen Norway; ^4^ Department of Pathology, Section for Neuropathology Oslo University Hospital Oslo Norway; ^5^ Department of Pathology, Faculty of Medicine University of Oslo Oslo Norway; ^6^ Department of Pharmacology, Medical Faculty University of Latvia Riga Latvia; ^7^ Department of Pathology Haukeland University Hospital Bergen Norway; ^8^ Department of Pathology St. Olavs Hospital Trondheim Norway; ^9^ Department of Pediatrics University Hospital of North‐Norway Tromsø Norway; ^10^ Faculty of Health Science The Arctic University of Norway Tromsø Norway; ^11^ Department of Pathology University Hospital of North‐Norway Tromsø Norway; ^12^ Department of Pediatrics St. Olavs Hospital Trondheim Norway; ^13^ Faculty of Medicine and Health Sciences, Department of Clinical and Molecular Medicine NTNU Trondheim Norway

**Keywords:** biobank, cancer, pediatric tumors, prospective, tissue collection

## Abstract

**Background:**

The rapidly expanding era of “omics” research is highly dependent on the availability of quality‐proven biological material, especially for rare conditions such as pediatric malignancies. Professional biobanks provide such material, focusing on standardized collection and handling procedures, distinctive quality measurements, traceability of storage conditions, and accessibility. For pediatric malignancies, traditional tumor biobanking is challenging due to the rareness and limited amount of tissue and blood samples. The higher molecular heterogeneity, lower mutation rates, and unique genomic landscapes, however, renders biobanking of this tissue even more crucial.

**Aim:**

The aim of this study was to test and establish methods for a prospective and centralized biobank for infants, children, and adolescents up to 18 years of age diagnosed with cancer in Norway.

**Methods:**

Obtain judicial and ethical approvals and administration through a consortium, steering committee, and advisory board. Develop pipelines including SOPs for all aspects in the biobank process, including collection, processing and storing of samples and data, as well of quality controlling, safeguarding, distributing, and transport.

**Results:**

The childhood cancer biobanking started at Oslo University Hospital in March 2017 and was from 2019 run as a national Norwegian Childhood Cancer Biobank. Informed consent and biological samples are collected regionally and stored centrally. Approximately 12 000 samples from 510 patients and have been included by January 1, 2021, representing a 96% consent and participation rate among our newly diagnosed patients.

**Conclusion:**

A well‐functioning nationwide collection and centralized biobank with standardized procedures and national storage for pediatric malignancies has been established with a high acceptance among families.

## 1 INTRODUCTION

Biobanks are repositories of biological samples with linked data. They are established to collect and harvest biological material during routine activities in an organized and standardized manner and therefore require pipelines for collection and facilities for storage of the biological specimens.^1^, ^2^ For pediatric oncology, the rapidly expanding era of “omics” (genomics, transcriptomics, proteomics, and metabolomics) and the impact on translational medicine is rendering the availability of biological material for research as increasingly crucial. Precise and effective treatment strategies based on genetic and molecular understanding of the diseases are increasingly being applied in pediatric cancer treatment, aiming to improve quality of life and resulting in further increase of survival ‐ cure rates. Hence, the investment in such professional collection and storage of biological material may in addition stimulate innovative biomedical development, enabling wide‐scale, and correlated clinical research.

Traditionally, tumor biobanking has been thematic and according to cancer type.^3^, ^4^ There are, however, strong arguments favoring the biobanking of pediatric malignancies as a collective entity. First, research shows that pediatric malignancies display distinct differences from adult cancers with higher molecular heterogeneity and lower mutation rates hence warranting separate research focus.^1^ Second, pediatric malignancies are rare, and the possibility for focus on unique genomic landscapes in pediatric malignancies naturally disappears in larger groups of samples where pediatric cases are greatly outnumbered. Thirdly, the limited amount of tissue and blood samples challenging all biobanking procedures is even more pronounced in children, where methods like needle biopsies for tissue sampling are necessarily applied.^5^ A national collection of samples for the whole entity of pediatric malignancies address these concerns by allowing for collection of larger sample sizes, and ensuring professional tracking, processing, storage of samples, and retrieval processes. Finally, there is an increasing demand for biological material for diagnostic purposes, clinical treatment protocols and research projects, making an organizational structure advantageous, enabling reuse of material and data generated from the material. This will lead to reduced consent fatigue, ensure good governance, increase collaboration activities among research groups both nationally and internationally, and reduce the risk of using the limited precious material for multiple and identical types of analyses.^6^

The Norwegian Childhood Cancer Biobank (NCCB) is a prospective biobank including infants, children, and adolescents from 0 to 18 years of age diagnosed with cancer in Norway.^7^ The overall aim of the NCCB is to stimulate and facilitate basic and translational research within childhood cancer through a national pipeline for biobanking tumor, microbiotic, and germline tissue at all regional childhood cancer departments and from all children with newly diagnosed cancer in Norway, providing the basis for advanced childhood cancer research on a national and international basis.

## 2 MATERIALS AND METHODS

### 2.1 Organizational structure

**FIGURE 1:**
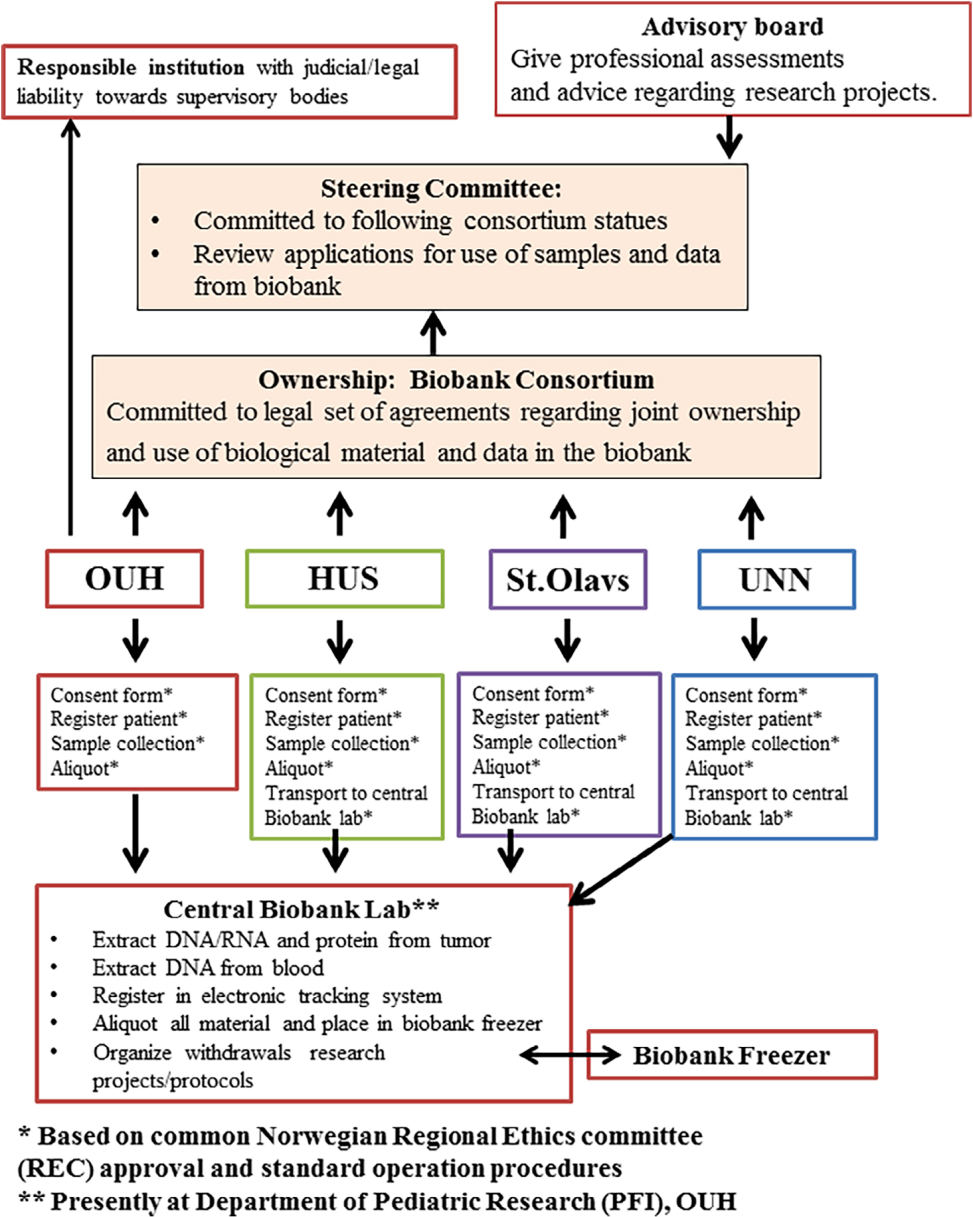
Structure of the Norwegian Childhood Cancer Biobank. The Norwegian Childhood Cancer Biobank is anchored in a national consortium and based on an agreement between the hospitals. Each consortium participant is responsible to legal set of agreements regarding joint ownership and use of biological material and data in the biobank. Oslo University Hospital is the responsible institution with the main juridical liability. Each university hospital has representatives in the steering committee, which is committed to follow the consortium statues and review application for use of samples and data from the biobank. In addition, it is established an advisory board, which gives their professional assessments and advice regarding research projects. The advisory board is composed of experienced researchers within the childhood cancer field and a user representative. The participants are invited by the steering committee to participate for 4 years, and the composition of the group aims to be a mix of basal medicine and clinical knowledge

The NCCB project was approved by the regional ethical committee (REC 2016/943–1) in 2016. The expansion towards national biobanking was organized through a national consortium (the National Childhood Cancer Biobank Consortium) consisting of representatives from the four regional hospitals Oslo University Hospital (OUH), Haukeland University Hospital (HUS), St. Olavs Hospital and University Hospital of Northern Norway (UNN) and user representative from the Norwegian Childhood Cancer Society. The biobank is administrated by a steering committee, and the applications for material recovery are reviewed by a scientific advisory board (Figure 1).

### 2.2 Inclusion by informed consent

Each regional hospital is responsible for including their respective patients to the NCCB. The patient group encompasses children admitted with suspected, newly diagnosed, or relapsed childhood cancer. For patients below age 16, both parents are required to sign the informed consent on behalf of the child. For patients between age 16 and 18, both patient and parents are required to sign. The NCCB use different consent forms to facilitate the information given based on the age of the child. All informed consent forms are approved by the ethical committee (REC 2016/943–1). Only patients with informed consent are included in the biobank. For each patient, a confidential biobank ID is generated. Data describing the biological material is registered in an electronic register (Table 1). The key link between the biobank ID and the personal ID is kept in a separate safety system, stored on servers for sensitive information at the four regional centers. The participants may withdraw their consent at any time, and because the parents are signing the consent on behalf of the child, a renewed signed consent from the participant is required when he/she reaches the age of 18. If the participant chooses to withdraw the consent from the biobank, all biological material will be destroyed, but research already performed will be kept.

**TABLE 1:** Clinical information collected for NCCB

Clinical information
Gender
Ethnicity*
ICD‐10 diagnosis and diagnosis category
Comorbidity*
Metastasis*
Age and date of diagnosis
Treatment protocol and start of treatment*
Other medications*
Family history (siblings, parents)*
Vaccination program*
Attended daycare (from what age)*

* Tentatively only collected at OUH.

### 2.3 Collection of samples

Biobank samples are collected after diagnostic material has been secured. Depending on the age and condition of the patient, samples are retrieved either awake or in general anesthesia. Optimally 4 ml blood is drawn into a K2EDTA VACUETTE (Greiner Bio‐One, Austria) and a serum clot activator VACUETTE (Greiner Bio‐One, Austria), heparinized bone marrow is collected into a Li‐heparin VACUETTE (Greiner Bio‐One, Austria) and spinal fluid is collected into a no additive VACUETTE (Greiner Bio‐One, Austria) for biobanking. In addition, the patients are asked to give noninvasive samples as buccal swabs (Whatman Sterile OmniSwab, GE Healthcare Life Sciences, USA) as well as urine, feces and hair where feasible (Table 2).

**TABLE 2:** Biological samples collected for NCCB

Biological samples
Blood
Bone marrow
Spinal fluid
Tumor tissue
Saliva
Urine*
Feces*
Hair*
Germline DNA
Tumor DNA
Tumor RNA

* Tentatively only collected at OUH.

### 2.4 Sample processing, aliquoting, and transport of samples

The blood samples are handled within one hour after sampling in the laboratory according to standard operational procedures (SOP). Whole blood from the EDTA tube (Greiner Bio‐One, Austria) is aliquoted into unique 0.5 ml matrix tubes (250 μl in each, Thermo Fisher Scientific, USA) and the rest of the blood is centrifuged at 2500× *g* to collect 250 μl aliquots of plasma and 500 μl buffy coat. Serum is collected after coagulation for minimum 30 min at room temperature and centrifugation at 2500× *g*. Urine, bone , and spinal fluid are aliquoted into 500 μl and 250 μl aliquots and frozen at −80°C.

Tumor biopsies are kept sterile and snap frozen preferably within 30–60 min after collection at the responsible Pathology Department. The tumor tissues are stored in 1.8 ml barcoded matrix tubes (Thermo Fisher Scientific, USA) and frozen at −80°C.

All samples are transported from the regional hospitals to the Department of Pediatric Research at OUH (Figure 1) at regular intervals. The samples are transported in approved styrofoam boxes filled with dry ice with parcel tracking and overnight express to minimize temperature changes and ensure optimal storage conditions.

All aliquots are registered in a safety approved electronic tracking system delivered by Laboratory Information Management System (LIMS) (LabWare, USA). The matrix barcode on the tube (Thermo Fisher Scientific, USA) labels each sample with an unique sample identifier, and the LIMS makes it possible to connect information such as SOPs, collection time, additive, and thaw count to the barcode. In addition, the system provides a storage hierarchy for tracking the aliquots for later analyses.

### 2.5 DNA/RNA extraction

DNA and RNA are extracted at the Department of Pediatric Research (OUH). Germline DNA from patients with solid tumors are extracted from whole blood using spin column technology with QIAamp DNA blood mini/midi kit (QIAGEN, Germany) and germline DNA from leukemia patients are extracted from buccal swabs (GE Healthcare Life Sciences, USA) with QIAamp Investigator Kit (QIAGEN, Germany) or complete remission bone marrow or blood by using QIAamp DNA blood mini/midi kit (QIAGEN, Germany).

Tumor tissue is sectioned and stained with hematoxylin and eosin to determine tumor percentage. DNA and RNA are extracted from tissue with >40% tumor using AllPrep DNA/RNA mini kit (QIAGEN, Germany).

Quality control is performed by measuring the optical density while DNA and RNA purity is determined based on the 260/230 and 260/280 ratio, using a NanoDrop spectrophotometer (ND‐1000, Thermo Scientific, USA). In addition, fluorescence measurements are used to determine the concentration and yield of DNA by a Qubit DNA BR assay (Thermo Fisher Scientific, USA) and RNA by a Qubit RNA BR Assay (Thermo Fisher Scientific, USA) on a Qubit 4 Fluorometer (Invitrogen, Thermo Fisher Scientific, USA). Based on the Qubit measurements, 500 ng DNA/RNA is aliquoted into six barcoded matrix tubes (Thermo Fisher Scientific, USA), registered in the LIMS system and stored at −80°C.

### 2.6 Storage

All biological samples and the extracted DNA and RNA samples are stored in monitored −80°C ultra‐freezers located in monitored storage facilities at the Norwegian Institute of Public Health. The freezers are connected to external alarm systems and temperatures are logged routinely to assure stable storage temperature and good preservation of the quality of the samples. The freezers are connected to an emergency electrical system to provide backup power and the facility provides backup freezers, which constitutes 10% of the total freezer capacity.

### 2.7 Quality assurance and quality control procedures

Each sample is handled according to SOPs, which are regularly reviewed and updated. Connected to every patient is a flowchart, which is filled out by responsible study nurse and authorized laboratory personnel. Time point of sampling and centrifugation is noted, in addition to cold ischemia time and freezing point for tumor tissue. Deviations from the SOPs are noted in the flowchart and registered in the LIMS. In addition to SOPs and the flowchart, NCCB has an ongoing study investigating changes in quality and quantity of DNA based on time to freeze and longtime storage of whole blood. These results will be presented in a coming manuscript.

## 3 RESULTS

**FIGURE 2:**
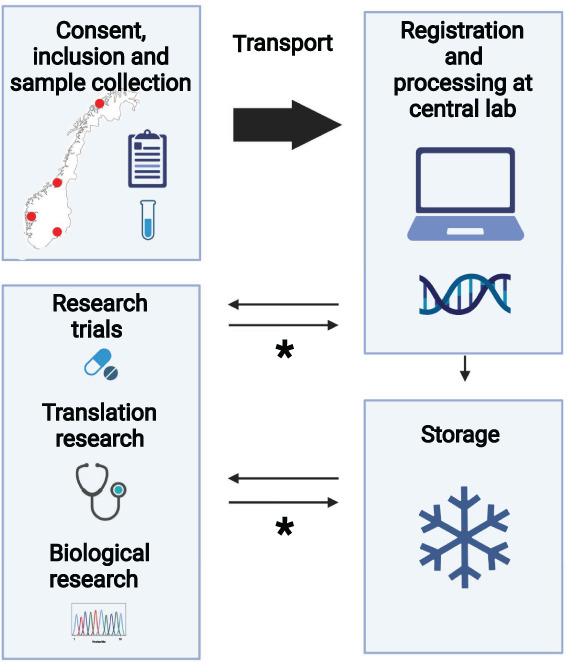
Norwegian Childhood Cancer Biobank work flow. Consent, inclusion, and sample collection according to Table 2 are performed at all four region hospitals. All samples are transported to the central lab at OUH for electronical registration and further processing. The samples are either stored at longtime storage or sent directly to research projects. *Surplus material are transported back to NCCB for reuse in research. Created with BioRender.com

Childhood cancer biobanking started at the OUH in March 2017 and expanded to include children on a national basis from January 2019 (Figure 2). The infrastructure of sample collection has been endorsed and supported at all four regional pediatric clinics, with the ambition of integrating sampling pipelines into the clinical ward as standard procedures. Tumor tissue, germline tissue (blood samples or buccal swabs), microbiotic tissue, and hair samples are all collected at time of diagnosis, in addition to blood samples and tumor tissue in case of relapsed disease. Health outcome information may be made available for all patients through linkage to the hospital journals or Norwegian national health registries upon approval by ethical committee.

### 3.1 Status to date

As of January 1, 2021, a total of 510 patients have been included and approximately 12 000 samples have been processed and stored in the biobank (Table 3). Of all patients invited to contribute to the NCCB biobank, 96% have consented to participate. Feasibility has been shown regarding collection of informed consent and biological samples locally, regular transportation of material to OUH, and central treatment of samples according to standardized procedures.

**TABLE 3:** Diagnoses of the first 500 patients collected for NCCB

Diagnoses	Percentage of patients
**Solid tumor**	**34**
Lymphoma	13
Wilms tumor	8
Neuroblastoma	7
Retinoblastoma	6
Rhabdomyosarcoma	5
Osteosarcoma	<5
Ewing sarcoma	<5
Germ cell tumors	<5
Hepatic tumors	<5
Other rare tumors	<5
**CNS**	**18**
Glioma	6
Medulloblastoma	<5
Astrocytoma	<5
Other tumors related to CNS	<5
**Leukemia**	**35**
Acute lymphocytic leukemia	29
Acute myeloid leukemia	5
Chronic myeloid leukemia	<5
**Other**	**13**
Other noncancer diagnoses	5
Nonmalignant tumors	5
Langerhans cells histiocytosis	<5
Myelodysplastic syndrome	<5
Anemia	<5
Enlarged lymph nodes	<5

*Note*: The bolded values represent the number of patients in the main tumor groups; solid tumors, CNS tumors and liquid tumors. Abbreviation: CNS, central nerve system.

**FIGURE 3:**
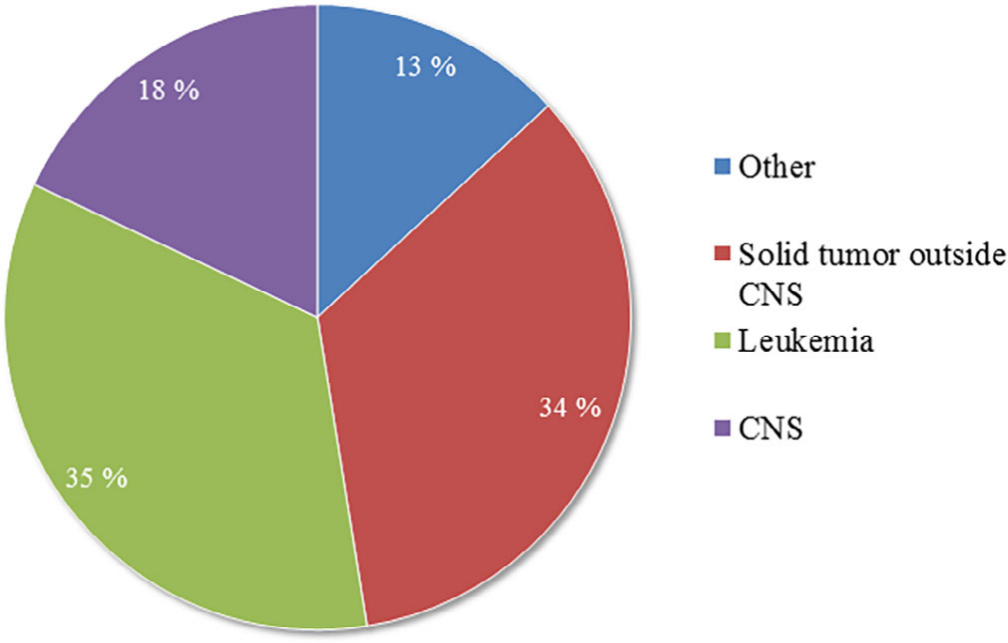
Diagnostic distribution of patients in the Norwegian Childhood Cancer Biobank. All included cancer diagnoses in NCBB are categorized into solid tumors, central nerve system (CNS) tumors, or leukemia, while nonmalignant diagnoses are placed in the category “other.” More information about the content of the different parts is listed in Table 3

Approximately 34% of the patients are diagnosed with leukemia, 18% have cancer related to the central nerve system (CNS), and 34% are diagnosed with solid tumors outside CNS. The remaining 13% are diagnosed with nonmalignant diagnoses (Figure 3). Diagnoses included in the groups and the number of patients diagnosed are presented in Table 3.

### 3.2 Impact on research

Material from about one third of all consented donors has been involved in eight different research projects. Ongoing studies are continuously published on our web page, including the name, responsible PI and a short description of the project (https://www.ous-research.no/home/childhoodcancerbiobank/Projects/19154). The involved projects cover a wide range from basic to translational research studies (e.g.^8^).

In addition, NCCB facilitates storage and coordination of transporting samples to international collaborative childhood cancer treatment studies. As biological research is increasingly becoming part of international childhood cancer treatment studies, and approximately 50% of our children are included in such studies, the NCCB has become an important part of the infrastructure needed to enable participation in these crucial studies.

## 4 DISCUSSION

Biobanking is an essential tool for medical research, in particular for diseases with few new cases per year. The ambition of the NCCB is to be a modern biobank, and actively facilitate and assist fundamental and applied clinical research by enabling access to biological material according to scientific requirements. The biobank aims to collect biological samples from all children and adolescents with newly diagnosed cancer in Norway. With a 96% inclusion rate, the invited participants have exhibited a very positive attitude regarding contribution towards the NCCB.

The importance of childhood cancer biobanking is becoming increasingly evident through other large‐scale pediatric biobank projects. The American Pediatric Cancer Genome project was launched in 2010 and demonstrated a significant difference in the spectrum of childhood cancers compared to adult cancers, as well as a large genomic heterogeneity between childhood cancers.^9^ The awareness of the differences between adult and childhood cancer, and the heterogeneity between pediatric cancer types, strengthens the importance of large‐scale collective biological sampling from this vulnerable and heterogeneous group of patients.

The NCCB aims to include all Norwegian pediatric patients with malignancies, as well as a varied range of samples per donor to cover various research fields and interests. This is a different approach from other Norwegian cancer biobanks such as the population based Janus Serum Biobank and the clinically based prospective Breast Cancer Biobank and Prostate Biobank, examples of adult biobanks related to cancer established in Norway. The prospective Breast Cancer Biobank and the Prostate biobank are cancer specific thematic biobanks consisting of donors from Western Norway and Oslo‐area respectively, while the Janus Serum bank collects blood tissue only from population based donors.^3^, ^10^, ^11^, ^12^ While these larger biobanks specialize on collecting and storing blood and/or tumor samples, the NCCB aims to collect as many relevant sample types as possible from our rarer number of cases. The collection of urine, feces, and hair opens the possibility to investigate environment exposures in childhood cancer care among others.

The inclusion rate of patients in the NCCB is extremely high, and represents the childhood cancer incidences in Norway given by the National cancer registry.^13^ A total of 35% of all participants included in NCCB are diagnosed with leukemia, which is in line with the expected national distribution of childhood cancer diagnoses based on numbers from 1985 to 2018 from the Norwegian Cancer Registry's 2018 Annual Report^13^ (Figure 3). The distribution of 80% of patients diagnosed with acute lymphocytic leukemia (ALL) and 20% diagnosed with acute myogenic leukemia within the leukemia group is also in agreement with the numbers from the Norwegian Cancer Registry Report.^13^

Another strength of the NCCB is the close collaboration between the different departments and among the different hospitals. Centralization of treatment and storage of samples has posed challenges of both logistic and collaborative nature, as all parts are aware of the value and scarcity of biological material. However, the engagement of pediatric oncologists, pathologists, nurses, and scientists that are clinically involved in the care and treatment of the pediatric patients, and the development of a national administrative steering group, made the national biobank endeavor possible. This teamwork of professionals that are close to the pediatric wards is also the basis for further translational projects, including the individualized therapy for relapsed malignancies in childhood study,^8^ using the logistics of inclusion and sampling provided by the NCCB.

From the beginning, the NCCB has chosen to open for sample release to ethically approved prospective studies, and to facilitate storage and release of research samples to clinical treatment protocols and add‐on studies. Projects approved by the Norwegian Regional Ethics committee (REC) may apply for material from NCCB by submitting an application form provided on our homepage (www.ous‐research.no/barnekreftbiobank/docs/Barnekreftbiobank Application for Data and Biological Material vs 1 61.docx and www.ous‐research.no/barnekreftbiobank/docs/Barnekreftbiobank Application for use of General Consent.docx). The NCCB steering committee evaluates and approves the submitted proposals based on scientific impact and demanded infrastructure and resources.

This strategy is based on our aim to facilitate collaborations and research within childhood cancer, and ensure that samples are being used in research. Furthermore, the NCCB has had a close collaboration with the Swedish Childhood Tumor Bank during the initial phase.^14^ The protocols of the NCCB, the Swedish Childhood Tumor Bank and other relevant childhood cancer biobanks show many similarities with collection of germline and tumor material, but may differ in terms of additional sampling and up front analysis. The NCCB assembles a very broad selection of collected materials, but has not prioritized to perform many up‐front analyses (i.e., sequencing).

SOPs have been developed to handle germline and tumor material similar to their protocols, and consequently, data from both biobanks could be merged to increase the number of samples for retrospective research in the future. This kind of international collaboration opens up new aspects ‐ and evolve the old ones ‐ for biomedical researchers, clinicians, and industrial partners. Contemporarily, biobanks tend to organize themselves into international networks to increase the impact of research activities. Biobanking and Biomolecular Resources Research Infrastructure was one of the first European research infrastructure projects funded by the European Commission in January 2011.^15^ Another significant organization is EuroBioBank, with 26 members and samples combined with data available in the online catalogue.^16^ The NCCB will pursue to establish novel collaborations with national and international networks in the future and to continue to promote research collaborations in national and Nordic working groups, such as the Nordic organization for Pediatric Hematology and Oncology.

A major challenge for a childhood cancer biobank is the heterogeneity between types of childhood cancer. This might necessitate collection of samples across different clinical units, pathology departments, and will involve many different disciplines. For example at OUH, CNS tumor patients are diagnosed at a different department than other childhood cancer patients, making standardized inclusion logistically more challenging. This may explain the lower inclusion rate of CNS patients (20% vs. the 26% patients expected according to the Norwegian Cancer Registry Report).^13^

The NCCB is operating in compliance with current biobanking standards, but has not yet been formally certified according to ISO standards as the accreditation system for biobanks is still under revision. It is planned to implement ISO 20387:2018 standards for biobanking with accreditation for the specific biobank procedures in the future. This will be a confirmation of the competence of the biobank and state appropriate quality of both biological samples and data collections.^17^

In conclusion, the NCCB provides a wide range of biological samples from all consenting childhood cancer patients in Norway, according to standardized procedures, ensuring proper quality and quantity of the stored material. In addition to collecting and storing samples for future research, the NCCB is actively supporting both translational projects as well as more basic research projects. All project using samples from NCCB are listed and described in further detail on the public webpage of NCCB (https://www.ous-research.no/barnekreftbiobank/).

## AUTHOR CONTRIBUTIONS

**Johanne Hermansen:** Conceptualization (equal); methodology (equal); writing &ndash; original draft (lead); writing &ndash; review and editing (equal). **Dorota Wojcik:** Conceptualization (equal); project administration (equal); writing &ndash; original draft (equal); writing &ndash; review and editing (equal). **Nina Robinson:** Methodology (equal); project administration (equal); writing &ndash; review and editing (equal). **Jens Pahnke:** Conceptualization (equal); project administration (equal); writing &ndash; review and editing (equal). **Hans Haugland:** Conceptualization (equal); project administration (equal); writing &ndash; review and editing (equal). **Ann‐Helen Jamtøy:** Conceptualization (equal); project administration (equal); writing &ndash; review and editing (equal). **Trond Flægstad:** Conceptualization (equal); project administration (equal); writing &ndash; review and editing (equal). **Hanne Halvorsen:** Conceptualization (equal); project administration (equal); writing &ndash; review and editing (equal). **Bendik Lund:** Conceptualization (equal); project administration (equal); writing &ndash; review and editing (equal). **Lars Bambusch:** Conceptualization (equal); methodology (equal); project administration (lead); writing &ndash; original draft (equal); writing &ndash; review and editing (equal). **Monica Munthe‐Kaas:** Conceptualization (equal); methodology (equal); project administration (lead); writing &ndash; original draft (equal); writing &ndash; review and editing (equal).

## CONFLICT OF INTEREST

The authors have stated explicitly that there are no conflicts of interest in connection with this article.

## ETHICS STATEMENT

Biobanking of biological material in the NCCB is based on informed consent, and the NCCB projects and infrastructure is supported by all four regional hospitals and approved by the Ethical Committee (REC 2016/943–1).

## Data Availability

Data sharing is not applicable to this article as no new data were created or analyzed in this study.
